# Static Crow’s Feet Treated with Voltaic Arc Dermabrasion (Atmospheric Plasma): Post-Operative Pain Assessment by Thermal Infrared Imaging

**DOI:** 10.3390/jcm10143074

**Published:** 2021-07-12

**Authors:** Antonio Scarano, Francesco Inchingolo, Domenico Amuso, Giuseppe Scogna, Roberto Amore, Felice Lorusso

**Affiliations:** 1 Department of Innovative Technologies in Medicine & Dentistry and CAST, University of Chieti-Pescara, Via dei Vestini 31, 66100 Chieti, Italy; giuseppescogna@hotmail.com; 2 Department of Interdisciplinary Medicine, University of Bari “Aldo Moro”, 70121 Bari, Italy; f.inchingolo@icloud.com; 3 Department of Techniques of Aesthetic Medicine and Wellness, University of Palermo, 90100 Palermo, Italy; d.amuso.estetica@gmail.com (D.A.); robertoamore@hotmail.com (R.A.); 4 Department of Innovative Technologies in Medicine & Dentistry, University of Chieti-Pescara, 66100 Chieti, Italy; drlorussofelice@gmail.com

**Keywords:** crow’s feet, skin rejuvenation, dermabrasion, voltaic arc dermabrasion, VAD, atmospheric plasma

## Abstract

Background: In the literature, several strategies have been described for the treatment of unaesthetic marks on the face resulting from the aging processes. The atmospheric plasma procedure is a non-invasive, inexpensive technique proposed for the rejuvenation of facial tissues. The aim of this study was to evaluate the performance of voltaic arc dermabrasion (VAD) for the treatment of static crow’s feet of the periorbital area. Methods: The crow’s feet of 135 patients (127 female and 8 male) were treated using the VAD technique. The perioperative skin temperature measurement was assessed using an Infrared Temperature sensor. The pain was measured using the Visual Analogic Score (VAS) at 1 week, 1 month and 1 year. The patient’s and surgeon’s satisfaction were assessed using the Global Aesthetic Improvement Scale (GAIS) at 1 month and 1 year from the procedure. The severity of the crow’s feet was rated using the Crow’s Feet Grading Scale (CFGS). Results: A complete epidermal healing of all the subjects treated was evident at 7 days. The atmospheric plasma technique showed an efficient treatment for the removal of the crow’s feet, with a good aesthetic outcome, high surgeon and patient satisfaction, without clinical complications. Conclusions: The atmospheric plasma technique can be a useful modality in the cosmetic as well as therapeutic treatment of crow’s feet.

## 1. Introduction

The aging of facial skin takes place gradually, over two to four decades, with little clinical evidence. Such regional anatomical variation has not always been acknowledged in the past, although it merits attention. The aging of the periorbital region plays an important role in a psychological and in a sociocultural way on the quality of life and is the first area that is exposed to ultra-violet radiation (UVR) and environmental damage, which may vary widely from individual to individual. The aging of this area of the face is speeded up also by repeated facial expressions; indeed, crow’s feet are accentuated by the contraction of the orbicularis oculi muscle that controls the closure of the eyelids. Periorbital wrinkles are a crucial component of facial aging that can be reduced with botulinum toxin treatment. Smoking can increase the normal aging process of the skin, contributing to the formation of wrinkles. During aging there is a change in the elasticity of the dermis with a reduction in the total amount of collagen [1]. Biochemically, the elastic fibers, which become tiny and fragmented, expand in a laminar shape between the collagen bundles with a reduction in the ratio of collagen type I to type III. There is an increase in skin pigmentation, and loss or redistribution of soft tissue volume and bone [2]. These changes are caused by atrophy and cutaneous ptosis and by the reduction in the craniofacial bones. Additionally, the muscles of facial expression increase the formation of wrinkles through boundaries of the deep mid facial fat compartments [3]. Indeed, midface bony elements such as maxilla, zygomatic, nose, mandible and teeth provide the support for all the overlying structures [4].

Different treatments have been proposed such as topical creams [5], botulinum toxin treatment [6], dermal fillers [7], chemical peels [8] and laser resurfacing [9]. In this study, the authors evaluated the clinical efficacy of voltaic arc dermabrasion (atmospheric plasma) for the treatment of static crow’s feet.

## 2. Materials and Methods

**Figure 1:**
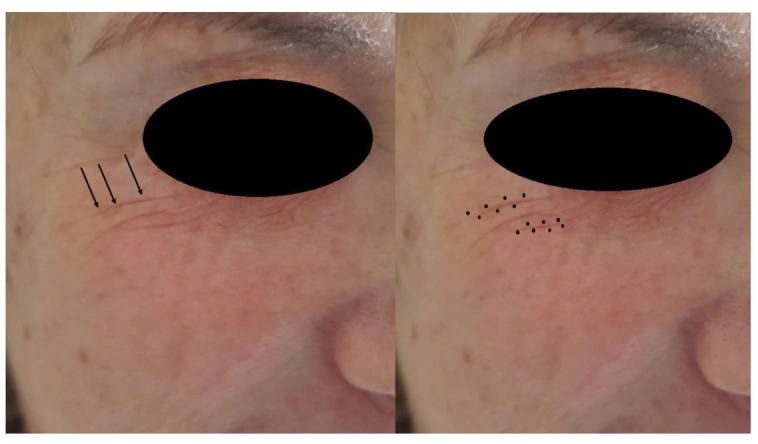
(**Left**)—Before treatment of the crow’s feet (arrows). (**Right**)—Individual tissue spots of excessive skin laxity are sublimated according to a triangular or zigzag distribution over the excessive skin, without penetrating into wrinkles.

**Figure 2:**
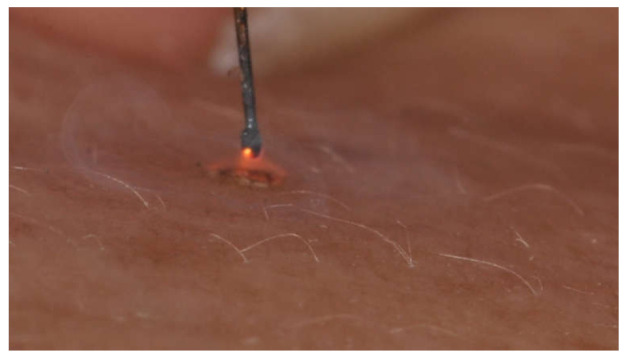
During plasma exeresis of crow’s feet with atmospheric plasma. The pin does not work if held in direct contact with the tissue to be treated, since it requires a small gap to be left for the generation of the plasma forming electric arc.

This retrospective study was conducted in accordance with the Declaration of Helsinki of 2013, which was adhered to strictly, and the additional requirements of Italian law, and was performed in a private office of Montesilvano, Bari and Modena (Italy). The authors treated the crow’s feet of 135 patients (127 female and 8 male) with the voltaic arc dermabrasion technique (Figure 1; Figure 2).

The inclusion criteria were periorbital lines in patients with a minimum age of 50. Twenty patients had Fitzpatrick skin type IV, ninety-two patients had type III and twenty-three patients had type II. In this study patients with severe periaorbital wrinkles were excluded and only patients with fine and moderate wrinkles were treated.

The exclusion criteria were previous periorbital surgery and unwillingness to complete the follow up. All patients received a complete head and neck examination at the initial visit and standardized photographs were taken. Postoperative visits were at 1 week and 1 month. At each visit, careful attention was paid to any complications or evidence of periorbital lines.

Keloids or connective tissue diseases are contraindications for undergoing this treatment as well as previous radiation therapy or scleroderma, which result in a reduction in adnexal structures, as there will be an absence of stem cells in the appendageal bulge, resulting in a reduction in postoperative re-epithelialization. The patients read the brochure that describes the possible complications and untoward effects, such as bruising or swelling and, after having discussed the risks, the benefits of and alternatives to periorbital rejuvenation, they each signed the informed consent form. Voltaic arc dermabrasion (VAD, Europe Medical s.r.l. Montesilvano (PE), Italy) was then employed to remove the keratinized layer from the periorbital area.

The treatment is minimally painful and only requires a local anesthetic, which was a Lidocaine 2.5%/prilocaine 2.5% EMLA cream (AstraZeneca S.p.A. Milan, Italy) that was gently applied on the lesions 30–60 min before the treatment. No anesthetic by infiltration was used.

The medical device consisted of a hand-held atmospheric pressure plasma jet, operating with an electrode discharge in atmospheric gas. The VAD device used was connected to a commercial 50 kHz high voltage alternating current power supply (3 kV, 2 mA) at 2 W.

The electrodes of the plasma device jetted the energy to the sides of the peri wrinkles, while the fingers of the non-dominant hand contracted and fixed the periorbital skin to accentuate the wrinkles (Figure 2).

**Figure 3:**
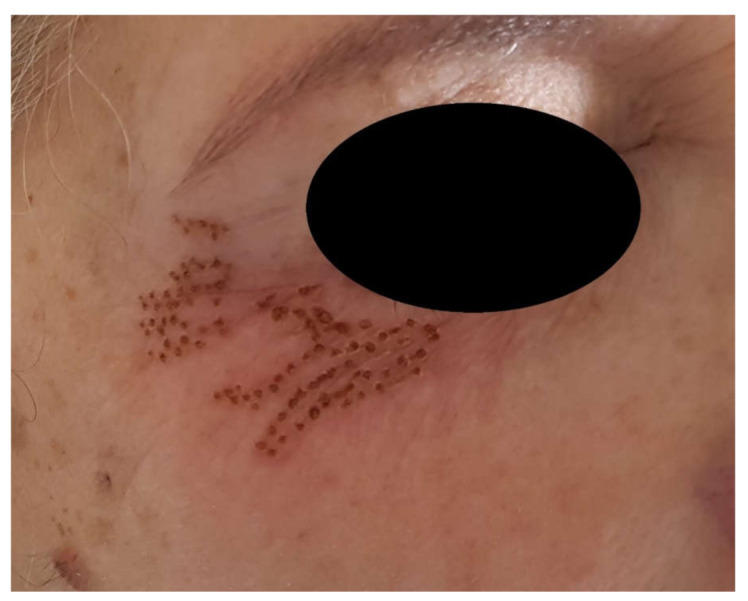
After treatment of the periorbital area. Appearance of a typical patient immediately after undergoing dermabrasion with the voltaic arc technique. Some degree of erythema is recorded immediately after the procedure.

The number of skin ablation points depended upon the skin type and the depth of the wrinkles (Figure 3). Applications have a duration of less than two seconds. Uniformity of treatment was achieved in most cases.

A first single peri wrinkle spotting was carried out with a non-overlapping and vaporizing voltaic arc. The resulting desiccated debris was then accurately removed with saline-soaked sponges. Subsequent passages gave a transient blanch that lasts from 10 to 15 s. The skin surface of the periorbital area then turns to a pink hue, showing a partially denatured papillary dermis. A hypoallergenic fluid foundation (Dermacol Cover, KRYOLAN GMBH, Berlin, Germany) was applied after the procedure to protect the area and to cover the carbonaceous layer residue of the treatment. The patients required no ulterior instructions and could go immediately back to their normal daily routine.

**Figure 4:**
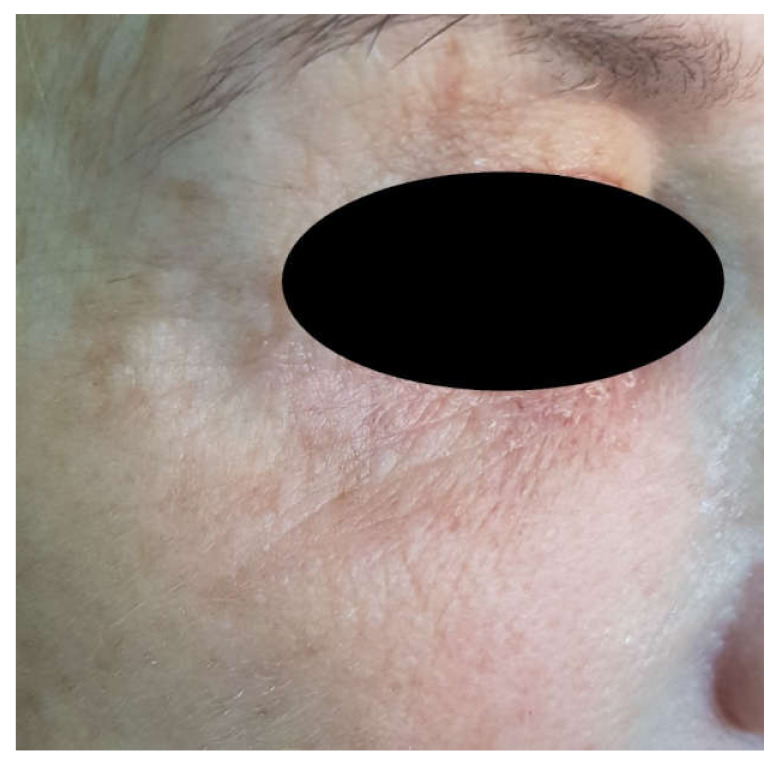
The periorbital area 1 month after treatment with an improved cosmetic outcome. There was no instance of pigmentary alteration or scarring as a result of treatment.

All patients received a single treatment of Voltaic arc dermabrasion technique for removing excess and laxity of the periorbital skin: in only one case was it necessary to repeat the treatment after 4 weeks (Figure 4). The patients returned for 1-week, 1-month and 1-year evaluations after the treatment. A joint investigator evaluated the results by means of clinical observation and a comparison of photographs of the periorbital areas taken pre- and post-treatment and at each follow-up visit.

### 2.1. Evaluation of Postoperative Healing

Postoperative healing was evaluated by means of comparative photographs, that permitted to measure the entity of erythema after 1 week and after 1 month; pain and surgeon and patient satisfaction were also recorded at the same time points.

Erythema (ER) was evaluated and classified into the following four categories: 1 (absent erythema), 2 (erythema extending < 1 mm), 3 (erythema extending < 2 mm) and 4 (intense erythema extending > 2 mm in the treated zone) [10].

Postoperative pain was scored by means of a 100-millimeter VAS from 0 (no pain) to 100 (worst pain imaginable). At the 1-week, 1-month and 1-year visit, standardized photographs were again taken, and the patient’s and the surgeon’s satisfaction were measured by means of a validated Global Aesthetic Improvement Scale (GAIS), as follows:

- Grade 5: Excellent (completely satisfied with the result);
- Grade 4: Very good (very satisfied with the result);
- Grade 3: Satisfactory (although a slight improvement is seen, an additional correction is required);
- Grade 2: Indifferent (no changes);
- Grade 1: Unsatisfied (the patient´s condition is worse than before the procedure).

Percentage change in wrinkles and change in subjective patient scores was compared between before and after treatment.

Treatment response was measured using the Crow’s Feet Grading Scale (CFGS) [11]. This is a photonumeric rating scale, in 5-points, which was applied to evaluate the severity of the crow’s feet. The scale ratings are 0 for no wrinkles, 1 for very fine wrinkles, 2 for fine wrinkles, 3 for moderate wrinkles and 4 for severe wrinkles.

### 2.2. Skin Temperature Measurements

Thermal surveys were carried out in a climate-controlled room (temperature: 22–24 °C, relative humidity: 50 ± 5%, with no direct ventilation into the mouth of patients). Periorbital temperature of the treated side was measured using a 14-bit digital infrared camera (FLIR SC660 QWIP, Flir Systems, Danderyd, Sweden). The parameters of acquisition applied to the measurement were 320 × 240 pixels focal plane array; 8–9 µm spectral range; 0.02 K noise equivalent temperature differences (NETDs); 50-Hz sampling rate; optics: germanium lens; f 20; and f/1.5. The camera distance was set at 0.50 m away from the mouth for optimal spatial resolution. Images were acquired at a rate of 10 images per second and subsequently re-aligned using an edge-detection based method, using in-house software. A video was taken, and the photos were extrapolated via dedicated software. Temperature changes in the periorbital were elaborated on the realigned thermal images. Thermographic data analysis was conducted using FLIR QuickReport v.1.2 (FLIR Systems Inc., North Billerica, MA, USA), which includes an instrument for obtaining the maximum, minimum and average temperature of the periorbital area.

### 2.3. Statistical Evaluation

The power analysis of the experimental design model was performed by dedicated statistical software (http://clincalc.com/stats/samplesize.aspx; accessed on 6 May 2019) to determine the optimal sample number of patients required for a statistical significance in the analyses of pain score, skin temperature, validated Global Aesthetic Improvement Scale (GAIS) and improvement in the wrinkles. A calculation model was used for dichotomous variables (yes/no effect) by putting the effect incidence at 20% for controls and 80% for treated areas, alpha was set at 0.05 and power at 90% associated with the null hypothesis that the size of the two groups was equal. The optimal number of patients for the investigation was 24.

Skin was considered as the statistical unit and the statistical analysis of the data was carried out using Statview software (SAS Institute, Cary, NC, USA). Continuous variables were considered by means of the number of observations, mean and standard deviation. The normality of the data statistics of the pain score, skin temperature and wrinkle improvement was evaluated using the Shapiro–Wilk test. The study data were compared between the two groups using the Mann–Whitney test, and the VAS and swelling reported by the groups were evaluated using the Friedman test, where a *p* value of <0.05 was considered statistically significant.

## 3. Results

**Figure 5:**
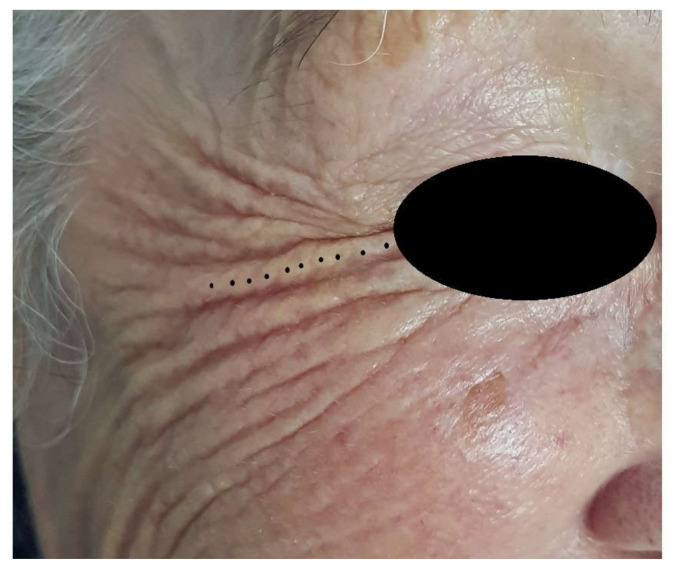
Before treatment of the crow’s feet.

**Figure 6:**
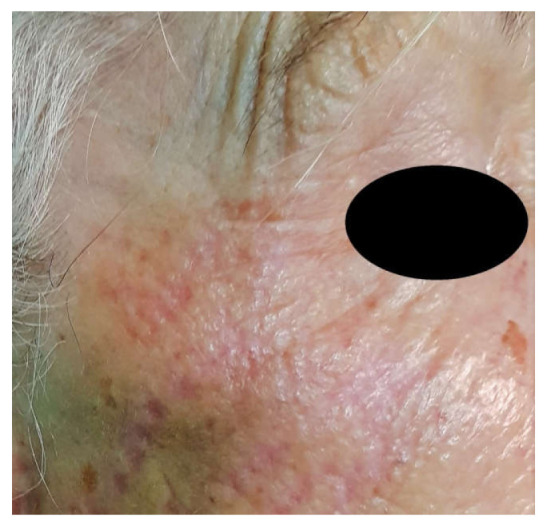
The periorbital area 1 month after the plasma exeresis with an improved cosmetic outcome.

**Figure 7:**
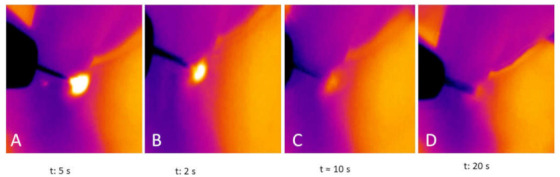
The temperature of periorbital skin decreased to basal measurements in 20 s. (**A**): initial irradiation timepoint (5 s); (**B**): after 2 s from the skin irradiation; (**C**): after 10 s from the skin irradiation; (**D**): after 20 s from the skin irradiation.

The mean age was 59.1 (51–67) years with a standard deviation of 5.1 years. Based on the VASs, very mild discomfort during plasma irradiation was reported in all the patients, with an average pain score of 3.45 ± 1.64. No pain or discomfort was recorded after plasma irradiation or during the follow-up after the procedure. Erythema, as a typical sign of heat application to the superficial skin, was present to a minimal extent only with an average 0.5 ± 0.1 immediately after the VAD irradiation and was decreased at 1 week (0.3 ± 0.1), no edema was recorded at 1 month or 1 year (Figure 4, Figure 5, Figure 6 and Figure 7).

Generally, no discomfort was experienced once the voltaic arc dermabrasion treatment was concluded, only five patients (14%) described feeling a moderate heat and tingling sensation. During the study period, 135 patients met the inclusion criteria, twelve of whom were smokers (27%).

**Figure 8:**
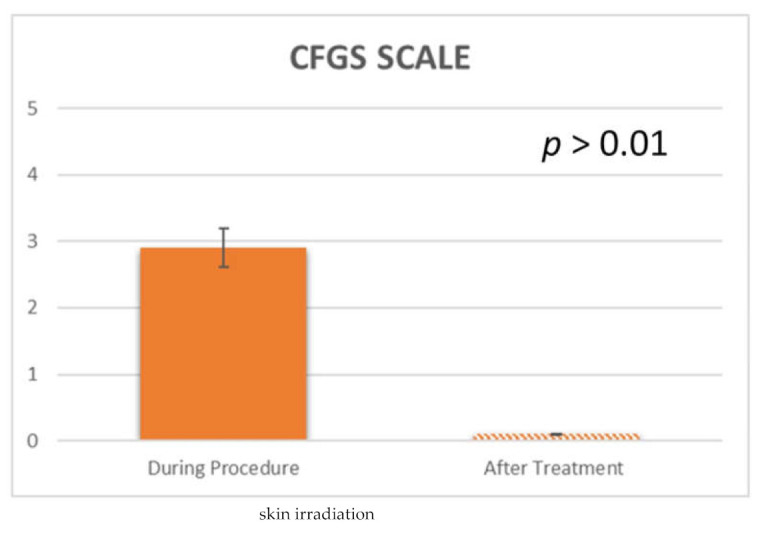
Crow’s Feet Grading Scale (CFGS) Score measured at the study follow-up.

**Figure 9:**
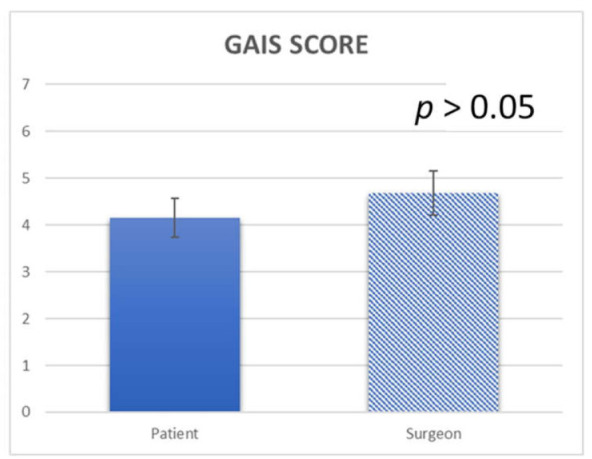
Satisfaction GAIS Score at 1 month and after 1 year from the treatment. A significant difference was detected between the two follow-up periods (*p* < 0.05).

During the first postoperative week, only two patients exhibited edema in the area, while at the 1-month follow-up examination, no edema was present. The results at one month after the treatment showed a mean patient satisfaction score of 4.15 ± 0.6, while the mean surgeon satisfaction score was 4.68 ± 0.5 (Figure 8). The results at one year after the treatment showed a mean patient satisfaction score of 3.25 ± 0.5, while the mean surgeon satisfaction score was 4.1 ± 0.3. No complications were observed, such as hyperpigmentation, hypopigmentation, ecchymosis, erythema, itching, pain, outbreaks of herpes, infectious processes nor scarring (Figure 4; Figure 6). A statistically significant difference was detected from baseline to 1 week and baseline to 1 month and 1 year (*p* = 0.006, *p* = 0.008 and 0.016, respectively) (Figure 8; Figure 9). Among the spots, there is a retraction of tissue with the approaching points flattening the wrinkle.

During the atmospheric plasma procedure, the average temperature of the skin was 270.3 ± 32.6 °C, while immediately after treatment it was 54.2 ± 14.4 °C (Table 1).

Regarding the overall subject satisfaction using the Crow’s Feet Grading Scale (CFGS) (11): before the VAD (atmospheric plasma) procedure, the average CFGS was 2.9 ± 0.2, while immediately after the treatment it was 0.1 ± 0.1 (Figure 8; Figure 9 and Table 2). No statistically significant difference was detected using CFGS from baseline to 1 week (*p* = 0.125) and a statistically significant difference after 1 month (*p* = 0.023) and after 1 year (*p* = 0.023).

The difference in the temperature before the procedure (basal measurement, 37.5 ± 2.6 °C) and that immediately after the atmospheric plasma irradiation was 17.3 ± 2.3 °C (Table 1).

The temperature decreased to basal measurements in 10 s in most of the patients and after 20 ± 0.32 s in all cases (Figure 7).

## 4. Discussion

In the present study, we used the atmospheric plasma technique for treating static crow’s feet. The investigators hypothesized that crow’s feet treated with atmospheric plasma can obtain good clinical results with less swelling and pain.

The outcome of this study shows that atmospheric plasma is an efficient technique for removing statical crow’s feet, with a short convalescence time and without complications. Overall surgeon and patient satisfaction were good. The postoperative epidermal re-epithelialization of the patients was complete by day 7, which is consistent with previous studies [12]. Bleeding was found to be minimal during vaporizing voltaic arc because a narrow zone of thermal injury seals most small vessels.

A previous study performed by this group showed that the atmospheric plasma technique improves the perioral area [13] and it was used with success for removing xanthelasma [14], upper eyelid blepharoplasty [15], benign skin lesions of the face [16] and telangiectasia of nose [17]. The application of atmospheric plasma is well tolerated clinically and allows for an effective improvement of skin laxity and rhytids. In this study, skin temperature changes were also investigated with an infrared camera and the data demonstrate evidence of a rapid increase in skin temperament with ablation of a thin layer of skin.

VAD was developed as a device for the cosmetic resurfacing of the facial skin and treatment of wrinkles of different areas of the face [12,16]. The temperature increase produces a skin contraction through the contraction of collagen. This phenomenon is well known in medicine and is used in aesthetic medicine for revengement of the upper eyelid, lip and preauricular wrinkles [18]. In our observation and as observed by others, a red/pink color after the first passage of atmospheric plasma indicates the removal of the epidermis. This technique is a procedure that uses atmospheric plasma to remove the upper layers of skin from the crow’s feet. In this treatment, collagen production is encouraged by the atmospheric plasma also heating the skin layers underneath, which stimulates the skin to heal in a smoother, more even appearance [12]. The arc voltaic is believed to be first described in 1801 by Ayrton, while the application for resurfacing facial skin was described by Scarano et al. in 2011 [12]. The VAD treatment uses ‘plasma’—the fourth state of matter after solid, liquid and gas. Atmospheric plasma is discharged in an arc that is characterized by a glow and a thermionic emission of electrons from the electrodes supporting the voltaic arc and the tip of the device dispenses energy.

The voltaic electric arc produces an ongoing electrical flow that, through a normally nonconductive medium such as air, produces a plasma that produces a visible light in the form of an electrical discharge with the highest current density. The plasma is an ionized gas that becomes highly charged and acts almost similarly to a tiny lightning bolt that effectively vaporizes or ‘sublimates’ the excess tissue, leaving a fine carbonized layer that disappears after two weeks. An arc of gas near atmospheric pressure is characterized by visible light emission with a high current density, and a high temperature (300–350 °C) that extends only on the surface tissue and not in depth. The current in the arc is supported by thermionic emission and a field emission of electrons at the tip [19]. The heat generates a plasma-induced contraction of collagen with skin shrinkage. The result is the flattening of the wrinkle. Traditional electrosurgery, on the other hand, uses high radiofrequency (RF) energy that rapidly vaporizes intracellular and extracellular fluids using the heat it generates (400–600 °C) [20,21], causing superficial tissue desiccation [22,23], but also going in depth into the tissue.

The plasma technique is an effective technique for removing wrinkles [14], while this technique, if operated by a well-trained surgeon, can produce excellent results on the appropriate patient. The keys for the correct use of the plasma technique are experience and understanding of the principles on which it is based, resulting in a sufficient resurfacing at the correct depth, while minimizing scar formation.

The described method has several advantages and disadvantages. The disadvantages include a technical learning curve requiring patience and a gentle touch. The advantages include a relatively quick and easy application once the learning curve is completed, an economic benefit, relatively simple equipment requirement (suited for large and small practices) and safe, effective results with no significant pigmentary changes (with a correct patient selection).

Another advantage of the plasma technique is that postoperative care is unnecessary [24]. Postoperatively, minimal edema resolves within several hours and the majority of patients can return to their normal daily activity immediately after the treatment and can even wear makeup if desired.

The plasma rapidly heats the skin, causing limited tissue ablation with minimal collateral damage [25] and rapid cooling of the tissues. Using a rabbit model, a study demonstrated how difficult it is to effectuate precise skin removal using a radiosurgical unit, while also controlling the tissue removal depth using the voltaic arc dermabrasion [12]. Epidermal healing is complete 7 days postoperatively and, at 30 days, neocollagenesis can be observed during histological analysis [13]. In the present research, we have not compared the use of laser vs. atmospheric plasma, but it seems that the use of the atmospheric plasma offers an excellent opportunity for the substantial removal of static crow’s feet.

This study showed that voltaic arc dermabrasion (atmospheric plasma) resulted in a slightly enhanced wound healing, while producing an almost bloodless field without the complications that can be observed in the use of botulinum toxin. In fact, a higher incidence of bruising was described in about 6% of treated subjects because the blood vessels were relatively more superficial and the skin was thin [26]. However, the botulinum toxin treatment represents the gold standard for the removal dynamic crow’s feet. In fact, the usual method for treating crow’s feet is botulinum toxin through multiple and repeated injections [27].

## 5. Conclusions

In conclusion, the outcomes of this study with plasma demonstrated that this good atmospheric plasma technique facilitates the removal of statical crow’s feet, is safe, effective and has a low downtime. It is characterized by no complications and minimal recurrence rates, with acceptable aesthetic results and was used with success for improving the appearance of the periorbital area.

## Figures and Tables

**Table 1:** Infrared temperature assessment at different experimental times.

Temperature	Perioperative Irradiation Point	After Irradiation Point	Perioperative Basal Measurement	Postoperative Temperature Difference
Mean (SD)	270.3 ± 32.6 °C	54.2 ± 14.4 °C	37.5 ± 2.6 °C	17.3 ± 2.3 °C
*p* value	*p* < 0.01		*p* < 0.01	

**Table 2:** Summary chart of Global Aesthetic Improvement Scale (GAIS) Satisfaction assessment and Crow’s Feet Grading Scale (CFGS) Scale at 1 month from the treatment.

GAIS Score			CFGS Scale	
Mean (SD)	Patient	Surgeon	During Procedure 2.9 ± 0.2	After Treatment 0.1 ± 0.1
	4.15 ± 0.6	4.68 ± 0.5		
*p value*	*p* > 0.05		*p* < 0.01	

## Data Availability

All experimental data to support the findings of this study are available contacting the corresponding author upon request. The authors have annotated the entire data building process and empirical techniques presented in the paper. The data underlying this article are not freely available by agreement with our partners to protect their confidentiality.
